# PEPT1 is essential for the growth of pancreatic cancer cells: a viable drug target

**DOI:** 10.1042/BCJ20210377

**Published:** 2021-10-21

**Authors:** Bradley K. Schniers, Devaraja Rajasekaran, Ksenija Korac, Tyler Sniegowski, Vadivel Ganapathy, Yangzom D. Bhutia

**Affiliations:** Department of Cell Biology and Biochemistry, Texas Tech University Health Sciences Center, Lubbock, TX 79430, U.S.A.

**Keywords:** glibenclamide, lactate, MMPs, pancreatic cancer, PEPT1, transmembrane H^+^ gradient

## Abstract

PEPT1 is a proton-coupled peptide transporter that is up-regulated in PDAC cell lines and PDXs, with little expression in the normal pancreas. However, the relevance of this up-regulation to cancer progression and the mechanism of up-regulation have not been investigated. Herein, we show that PEPT1 is not just up-regulated in a large panel of PDAC cell lines and PDXs but is also functional and transport-competent. PEPT2, another proton-coupled peptide transporter, is also overexpressed in PDAC cell lines and PDXs, but is not functional due to its intracellular localization. Using glibenclamide as a pharmacological inhibitor of PEPT1, we demonstrate in cell lines *in vitro* and mouse xenografts *in vivo* that inhibition of PEPT1 reduces the proliferation of the cancer cells. These findings are supported by genetic knockdown of PEPT1 with shRNA, wherein the absence of the transporter significantly attenuates the growth of cancer cells, both *in vitro* and *in vivo*, suggesting that PEPT1 is critical for the survival of cancer cells. We also establish that the tumor-derived lactic acid (Warburg effect) in the tumor microenvironment supports the transport function of PEPT1 in the maintenance of amino acid nutrition in cancer cells by inducing MMPs and DPPIV to generate peptide substrates for PEPT1 and by generating a H^+^ gradient across the plasma membrane to energize PEPT1. Taken collectively, these studies demonstrate a functional link between PEPT1 and extracellular protein breakdown in the tumor microenvironment as a key determinant of pancreatic cancer growth, thus identifying PEPT1 as a potential therapeutic target for PDAC.

## Introduction

Pancreatic ductal adenocarcinoma (PDAC) is one of the most lethal forms of cancer with a 5-year overall survival rate of 7% [1–6]. PDAC is currently the third leading cause of cancer-related deaths in the U.S., trailing only lung and colorectal cancer, and is projected to outpace colorectal cancer to become the second leading cause of cancer-related deaths by 2030 [2,6]. With such a bleak prognosis, there is a need for new, hitherto untested, therapies. Recognizing that maintenance of amino acid nutrition in cancer cells is obligatory for the rapid tumor growth, several studies focused on amino acid transporters as a key component of this phenomenon and hence potentially therapeutic targets [7,8]. Peptide transporters belonging to the SLC15 family could also support amino acid nutrition by providing di- and tripeptides to the cells, but little is known on this group of transporters for their role in cancer. PEPT1, a proton-coupled peptide transporter, is expressed primarily in small intestinal epithelium and is known to transport over 8 000 di- and tripeptides as well as peptidomimetic drugs [9–13]. The other three members in the SLC15 family include PEPT2, PHT1, and PHT2. PEPT2 is predominantly expressed in the kidneys; though very similar to PEPT1 in terms of function and substrate selectivity, PEPT2 has much higher substrate affinity than PEPT1. PHT1 and PHT2, unlike the other two proteins, transport histidine and only a limited number of di- and tripeptides [9].

Though PEPT1 is expressed primarily in the small intestine under normal physiological conditions, there is unequivocal evidence implicating its role in pathological conditions such as IBD in colon [14,15]. Studies have shown that PEPT1 promotes the growth and progression of CAC [16] and deletion of PEPT1 prevents the induction of DSS-induced colitis in mouse models. In fact, PEPT1 has been found to be up-regulated in biopsies of human colorectal cancer [16,17], gastric cancer [18], fibrosarcoma cells [19], hepatocarcinoma cells [20], cholangiocarcinoma cells [21], prostate cancer [22], and in some pancreatic cancer cell lines [23]. Nonetheless, except for the study in CAC [16], all other studies have shown the up-regulation of PEPT1 in cancer or cancer cells but have not investigated its role in cancer cell proliferation in any detail.

There is very little in the published literature on the association between PEPT1 and pancreatic cancer. It is known that PEPT1 is up-regulated in pancreatic cancer cell lines and that its inhibition suppresses the growth of cancer cells, but these findings were only limited to a few cell lines [23,24]. In the present study, we characterized the expression profile of PEPT1 in a large panel of PDAC cell lines and PDXs, and also monitored its transport function. Using pharmacological inhibition as well as genetic knockdown, we demonstrated the tumor-promoting role of PEPT1 in cancer cell lines *in vitro* and in mouse xenografts *in vivo*. Furthermore, we investigated the potential pathways for generation of peptide substrates for PEPT1 in the tumor microenvironment and the role of tumor-derived lactic acid in this phenomenon, thus linking the tumor-specific Warburg effect to PEPT1-mediated amino acid nutrition in cancer cells.

## Materials and methods

### Cell lines and cell culture

HPDE, a human pancreatic ductal epithelial cell line, was kindly provided by Dr. Ming Tsao, Ontario Cancer Institute (Toronto, Canada), and maintained in HPDE complete culture medium according to recommendations by Kerafast (Boston, MA). hTERT-HPNE, another human pancreatic ductal epithelial cell line, as well as human pancreatic cancer cell lines AsPC-1, BxPC-3, Capan-1, Capan-2, CFPAC-1, HPAF-II, MIA PaCa-2, PANC-1, Panc 10.05, and SU.86.86 were obtained from ATCC. All cells, except HPDE, were maintained in the respective media suggested by the ATCC (DMEM, RPMI-1640, or Iscove's DMEM), with 10% FBS, 100 U/ml penicillin, and 100 U/ml streptomycin. Cells were cultured at 37°C in a humidified atmosphere containing 5% CO_2_. All cell lines have been routinely tested for mycoplasma contamination using the Universal Mycoplasma Detection Kit obtained from ATCC. Additionally, all cell lines were received from ATCC with certification of each individual cell line within 2 years of each experiment. The patient-derived xenografts (PDXs) were provided by the Texas Cancer Cell Repository (www.TXCCR.org).

### Chemicals and antibodies

Glibenclamide (G0639), Gly-Phe (G2752), and Gly-Sar (G3127) were purchased from Millipore Sigma. Glycyl-L-proline (Gly-Pro, 405935) was purchased from Frontier Scientific. Puromycin dihydrochloride (A11138), Pierce Western Blot reagents, and Applied Biosystems qPCR reagents were purchased from ThermoFisher Scientific.

The antibodies specific for hSLC15A1 and hSLC15A2 were generously supplied by David E. Smith, College of Pharmacy, University of Michigan. Other antibodies used were: anti-β-actin (Santa Cruz Biotechnology, sc-47778), anti-LAMP-1 (Abcam, ab25630), anti-MMP9 (Cell Signaling, D6O3H), anti-MMP13 (Cell Signaling, E4W3T), anti-MMP16 (Abcam, ab73877), anti-DPP4/CD26 (Cell Signaling, D6D8K), goat anti-rabbit IgG (Bio-Rad, 170-6515), goat anti-mouse IgG (Bio-Rad, 170-6516), goat anti-mouse IgG alexa fluor 488 (ThermoFisher, A-11029), goat anti-rabbit IgG alexa fluor 594 (ThermoFisher, A-11037), goat anti-rabbit IgG alexa fluor 488 (Life Technologies Corp, A11008), goat anti-mouse IgG alexa fluor 647 (Life Technologies Corp., 647), and ProLong Diamond Antifade Mountant with DAPI (Life Technologies Corp., P36962),

### Plasmids and transfection

All PEPT1 shRNA variants (TRCN0000043298, TRCN0000043299, TRCN0000043300, TRCN0000043301, TRCN0000043302) were purchased from Millipore Sigma. pLK0.1 puro (8453) was purchased from addgene. PLP1, PLP2, and VSVG (Invitrogen, K497500) were purchased from Fisher Scientific. Lipofectamine 3000 (L3000015) was purchased from ThermoFisher Scientific.

For plasmid transfection, SU.86.86 cells were plated to form a 30% to 50% confluent culture. The 293FT cells were transfected using Lipofectamine 3000, according to the ThermoFisher protocol. The shRNA viruses were harvested from 293FT cells after 42 h and applied to SU.86.86 cells after filtration using 0.45 µm syringe filter. Viral medium was replaced with complete medium after 16 h, and cells were maintained in complete medium for 24 h. After 24 h, cells underwent selection for 10–14 days with 0.5 µg/ml puromycin added to the medium. Thereafter, all transfected cell lines were cultured in complete medium with 0.5 µg/ml puromycin. shRNA knockdown of PEPT1 was confirmed by qPCR, western blot analysis, and [^3^H]Gly-Sar uptake assay to assess PEPT1 mRNA, protein expression, and transport function.

### qPCR and western blot

RNA isolation, cDNA synthesis, and qPCR were performed as previously described [25]. The following PCR primers were used: PEPT1/SLC15A1 forward: 5′-CTCCCAATGTTCTGGGCCTT-3′ and reverse: 5′-CGTTCACGGTCTGCATCTGA-3′; PEPT2/SLC15A2 forward: 5′-ATCAGCAGGGTTCACGATGG-3′ and reverse: 5′-CCACACTTGGAGACCAGACG-3′. Cell line and PDX protein lysates were prepared, and western blot analysis was performed, as previously described [25]. In short, 30 µg of protein was loaded per lane in each of the western blots and electrophoretically separated on 10% SDS–Page gels. All proteins were transferred at 4°C overnight at 20 V to a nitrocellulose membrane (Bio-Rad), blocked with 5% Blotting Grade Blocker (Bio-Rad) at room temperature for 90 min, then incubated with primary antibody at 4°C overnight. All primary antibodies were diluted 1000-fold, with the exception of anti-β-actin, which was diluted 10 000-fold. After washing with TBST, the membrane was incubated for 1 h at room temperature with correlating secondary antibodies at a dilution of 3000-fold, washed in TBST again, then incubated for 5 min in Pierce ECL solution (ThermoFisher), before being visualized with either autoradiography films or an Azure Biosystems c300 imager.

### Radiolabeled Gly-Sar uptake assay

PEPT1 and PEPT2 functionality was analyzed by [^3^H]Gly-Sar (Moravek Inc., CA) uptake assay, which was performed as previously described [26]. The composition of the uptake buffer was 25 mM Mes/Tris (pH 5.5) or 25 mM Hepes/Tris (pH 7.5), 140 mM NMDG-chloride, 5.4 mM KCl, 1.8 mM CaCl_2_, 0.8 mM MgSO_4_ and 5 mM glucose. Cells were incubated with [^3^H]Gly-Sar in the uptake buffer for 15 min, before being washed with cold uptake buffer to terminate uptake. Cells were then lysed and transferred to counting vials for determination of radioactivity. For the kinetics studies, Gly-Sar uptake was measured at increasing concentrations of Gly-Sar (1–3160 µM) in AsPC-1 cells using [^3^H]Gly-Sar as the tracer during uptake measurements. The kinetic parameters (Michaelis constant *K*_t_ and maximal velocity *V*_max_) were determined using the computer program GraphPad Prism version 8.4.3. These determinations were made by non-linear regression analysis and the values were confirmed by linear regression analysis according to the Eadie–Hofstee transformation of the Michaelis–Menten equation. The effects of glibenclamide on PEPT1 function were studied using [^3^H]Gly-Sar uptake in Capan-1, MIA PaCa-2, and SU.86.86 cells in the presence of 100 µM glibenclamide. Likewise, a dose-response relationship for the inhibition of [^3^H]Gly-Sar uptake by glibenclamide was performed using increasing concentrations of glibenclamide (0.1–100 µM) to determine the IC_50_ value in Capan-1 and SU.86.86.

### Immunofluorescence

Immunofluorescence studies were performed as previously described [25]. In short, cells were grown to 60% confluency in chamber slides and stained with rabbit anti-PEPT1 or rabbit anti-PEPT2, and mouse anti-LAMP1 overnight at 4°C. Cells were then washed and stained with secondary antibodies (goat anti-rabbit, goat anti-mouse) conjugated with Alexa Fluor 488, 594, or 647, followed by washing and mounting with ProLong Diamond Antifade Mountant with DAPI. All images were captured using a Carl Zeiss LSM510 Meta upright confocal microscope.

### Colony formation assay

Colony formation assays were performed as previously described [25]. Control SU.86.86 and SU.86.86/shRNA-PEPT1 cells were first grown in complete culture media without additives; cells were then grown in three separate conditions, with glibenclamide added in a dose responsive manner: complete culture media, complete culture media with Gly-Pro added, or complete culture media with Gly-Pro added and pH adjusted to pH 6.5. After being allowed to form colonies for 2 weeks, cells were fixed, stained, and evaluated as previously described, using Enhanced Gram Crystal Violet (Remel).

### Lactate treatment and amino acid restimulation

PDAC cell lines SU.86.86 and AsPC-1 were used to determine effects of treatment with lactate, mimicking tumor extracellular environment. Cells were treated with sodium lactate in an incrementally increasing amount of time (30 min–15 h). Total RNA was harvested after treatment for qPCR analysis. qPCR was performed with MMP9, MMP13, MMP16, and DPPIV. Cell lines Capan-1 and SU.86.86 were utilized to determine regulatory aspects of amino acid deprivation and restimulation. Cells were cultured in amino acid- and serum-free medium for either 12 or 18 h, followed by restimulation with 20 mM phenylalanine, 20 mM lysine, 10 mM Gly-Pro, 10 mM Gly-Sar, or 10 mM Gly-Phe. qPCR and [^3^H]Gly-Sar uptake were performed as described. Since SU.86.86 could not sustain growth in culture media lacking FBS, 1% FBS was added to amino acid-free RPMI for all SU.86.86 deprivation experiments.

### Murine xenograft experiments

Male and female athymic nude mice (4-week-old) were purchased from Jackson Laboratories and housed under standard conditions. Control SU.86.86 and SU.86.86/shRNA-PEPT1 cells were injected subcutaneously into the right flank (3 × 10^6^ cells), using IACUC approved restraint with no anesthetic. SU.86.86 (5 × 10^6^ cells) and Capan-1 (2 × 10^6^ cells) were injected subcutaneously into both left and right flanks. All cells were suspended in serum-free media and Matrigel (1 : 1 ratio), with 100 µl of suspension being injected to each mouse. Mice injected with SU.86.86 and Capan-1 cells were treated by oral gavage daily, with either placebo (corn oil with 10% DMSO) or 166 mg/kg glibenclamide (corn oil with 10% DMSO containing glibenclamide). Tumor size was measured biweekly with caliper, with tumor volume calculated using the formula (width^2^ × length)/2. Tumors were allowed to grow for 21–30 days; mice were then killed via isoflurane induction and tumors harvested following the approved IACUC protocol. RNA and protein were prepared from the tumor tissue for qPCR and western blotting to determine expression levels of PEPT1 in shRNA tumors compared with control SU.86.86 cells. All murine experiments were performed in the Laboratory Animal Resource Center at Texas Tech University Health Science Center in Lubbock, TX.

### Statistical analysis

Statistical analysis and graphs were performed using GraphPad Prism 8.4.3. Results are expressed as mean ± SEM. All experiments were repeated thrice unless otherwise specified. Statistical significance was determined by unpaired Student's *t*-test.

## Results

### PEPT1 is up-regulated in PDAC cell lines and PDXs

**Figure 1.**
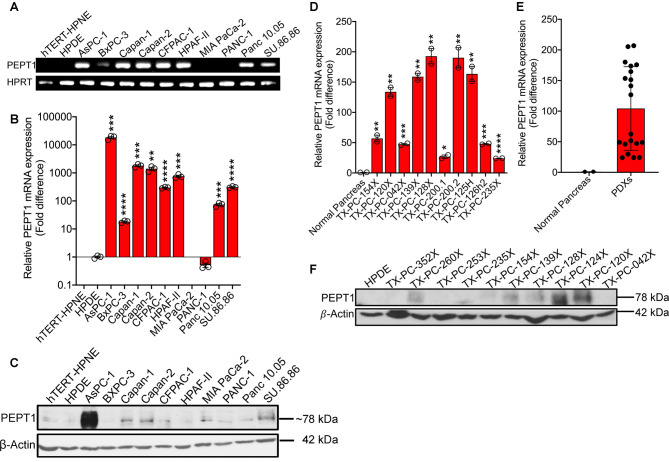
PEPT1 is up-regulated in PDAC cell lines and PDXs. (**A**) Regular RT-PCR showing mRNA expression of PEPT1 in hTERT-HPNE and HPDE (normal pancreatic epithelial cell lines) and 10 PDAC cell lines. HPRT was used as an endogenous control. (**B**) Real-time PCR showing relative PEPT1 mRNA expression in hTERT-HPNE cell line and 10 PDAC cell lines. (**C**) Western blotting showing PEPT1 protein expression in hTERT-HPNE and HPDE cell lines and 10 PDAC cell lines. β-Actin was used as an endogenous control. (**D**) Real-time PCR showing relative PEPT1 mRNA expression in normal pancreas and 10 patient-derived xenografts (PDXs). (**E**) PEPT1 mRNA expression in pooled PDXs. (**F**) Western blotting showing PEPT1 protein expression in normal pancreas and 10 patient-derived xenografts (PDXs). Data are given as mean ± SEM. * *P* < 0.05, ** *P* < 0.01, *** *P* < 0.001, **** *P* < 0.0001.

Although previous studies have shown PEPT1 up-regulation in pancreatic cancer, these studies were limited to very few cell lines. Therefore, we first wanted to check the expression profile of PEPT1 not only in a larger panel of PDAC cell lines but also in PDXs. We first performed a regular RT-PCR utilizing two normal cell lines (hTERT-HPNE and HPDE) and ten PDAC cell lines (AsPC-1, BxPC-3, Capan-1, Capan-2, CFPAC-1, HPAF-II, MIA PaCa-2, PANC-1, Panc 10.05, and SU.86.86). All of the PDAC cell lines tested, with the exception of MIA PaCa-2 and PANC-1, demonstrated a significant up-regulation of PEPT1 at the mRNA level (Figure 1A). Both the normal cell lines expressed negligible PEPT1. We corroborated these data using quantitative real-time PCR and showed up-regulation in PEPT1 mRNA 10–10 000-fold in the majority of the PDAC cell lines tested, relative to hTERT-HPNE (Figure 1B). Western blotting data convincingly showed that the up-regulated mRNA expression correlated with PEPT1 protein expression (Figure 1C). We further validated our cell line data using PDX samples. PEPT1 mRNA was significantly up-regulated in all the PDXs tested (Figure 1D) with an average fold increase in 100, compared with the normal human pancreas (Figure 1E). Likewise, majority of the PDXs also showed an increase in PEPT1 protein (Figure 1F). Taken together, these data indicated that PEPT1 is significantly up-regulated in pancreatic cancer at both mRNA and protein levels, with little or no expression in the normal pancreas.

### PEPT2 is also up-regulated in PDAC cell lines and PDXs

**Figure 2.**
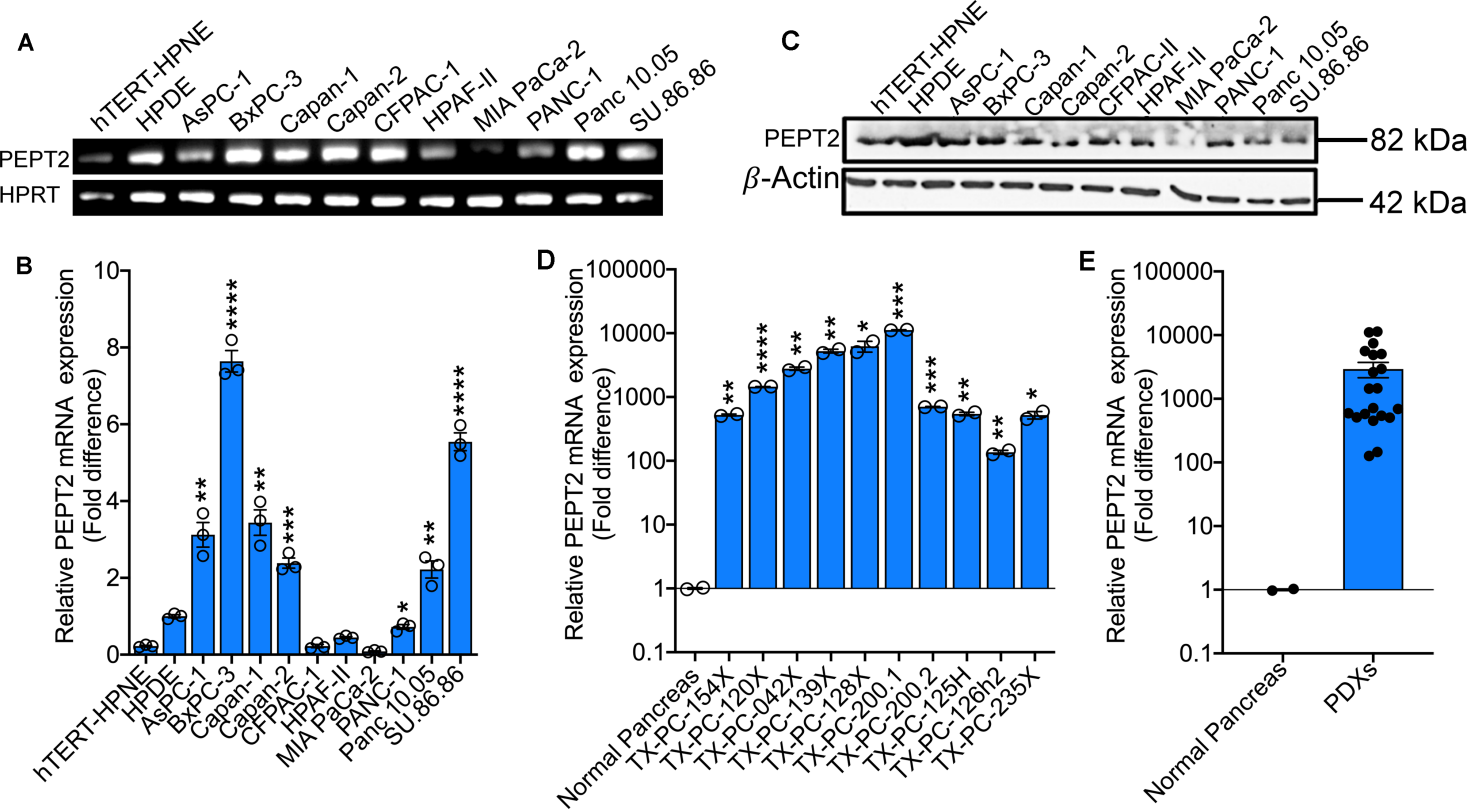
PEPT2 is up-regulated in PDAC cell lines and PDXs. (**A**) Regular RT-PCR showing mRNA expression of PEPT2 in hTERT-HPNE and HPDE cell lines and 10 PDAC cell lines. HPRT was used as an endogenous control. (**B**) Real-time PCR showing relative mRNA expression of PEPT2 in hTERT-HPNE cell line and 10 PDAC cell lines. (**C**) Western blotting showing PEPT2 protein expression in hTERT-HPNE and HPDE cell lines and 10 PDAC cell lines. β-Actin was used as an endogenous control. (**D**) Real-time PCR showing relative mRNA expression of PEPT2 in normal pancreas and 10 patient-derived xenografts (PDXs). (**E**) PEPT2 mRNA expression in pooled PDXs. Data are given as mean ± SEM. * *P* < 0.05, ** *P* < 0.01, *** *P* < 0.001, **** *P* < 0.0001.

Since PEPT2 is functionally very similar to PEPT1, we were curious to check its expression status in PDAC cell lines and PDXs. For this, we performed a regular RT-PCR and real-time PCR utilizing two normal cell lines (hTERT-HPNE and HPDE) and ten PDAC cell lines (AsPC-1, BxPC-3, Capan-1, Capan-2, CFPAC-1, HPAF-II, MIA PaCa-2, PANC-1, Panc 10.05, and SU.86.86). RT-PCR data showed an up-regulation of PEPT2 in almost all the cell lines tested (except MIA PaCa-2) compared with the normal hTERT HPNE (Figure 2A). However, HPDE, another normal cell line, did express PEPT2. Similar results were seen in real-time PCR (Figure 2B). PEPT2 protein was also expressed as evident from western blotting data, but there was no difference in the protein levels between the normal and PDAC cell lines (Figure 2C). Similar results were also seen in PDXs, with results establishing PEPT2 mRNA levels of 100-fold or higher in PDXs as compared with normal pancreas (Figure 2D,E).

### PEPT1, but not PEPT2, is functional in PDAC cell lines

**Figure 3.**
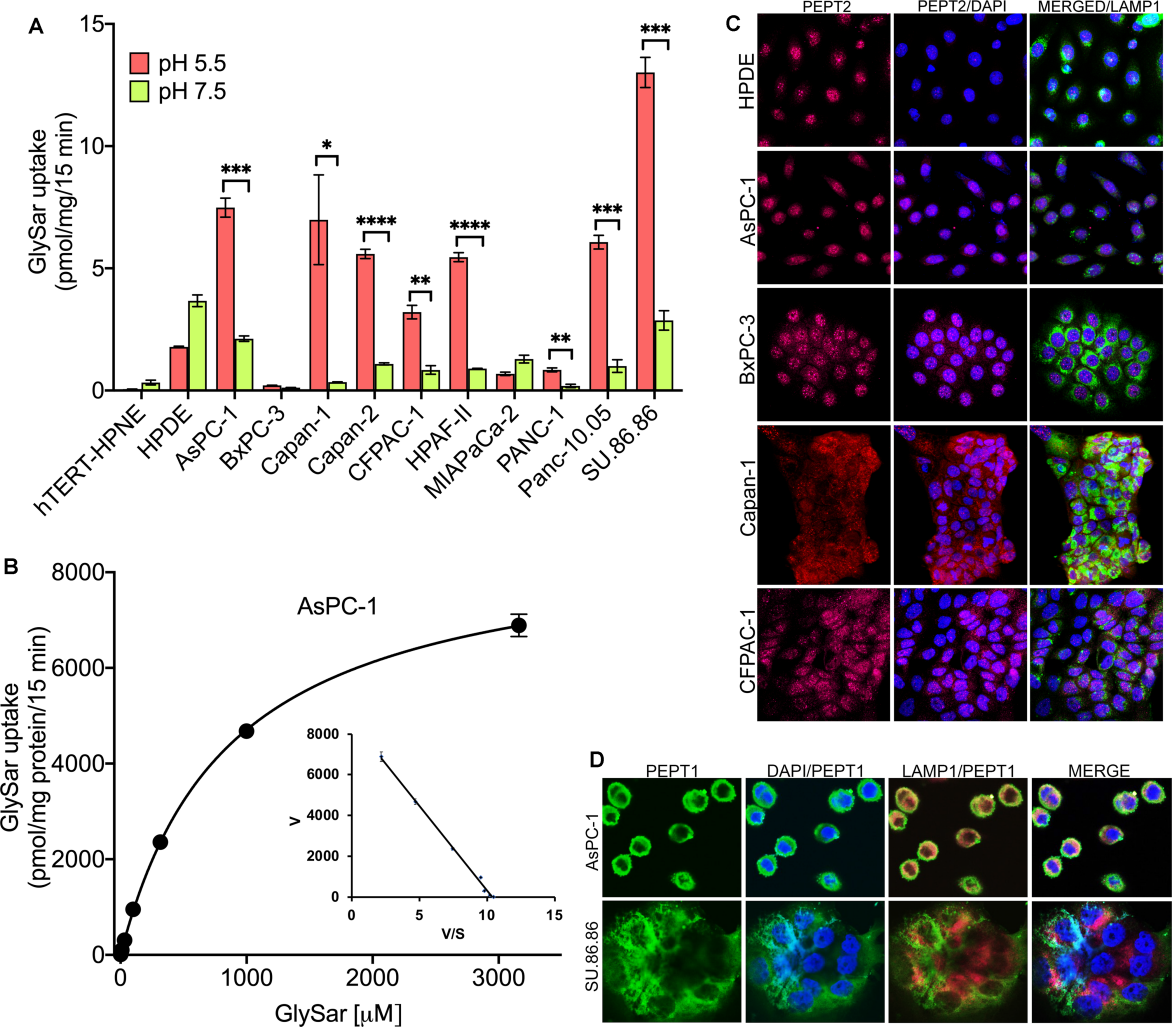
PEPT1, but not PEPT2, is functional in PDAC cell lines. (**A**) [^3^H]-Gly-Sar uptake showing functional uptake of the dipeptide in normal pancreatic cell lines hTERT-HPNE and 10 PDAC cell lines at pH 5.5 and 7.5. (**B**) [^3^H]-Gly-Sar kinetics in the presence of increasing concentration of unlabeled Gly-Sar in AsPC-1 cells. (**C**) Immunocytochemical detection of PEPT2 (red) in PEPT2-positive normal pancreas cell line (HPDE) and PDAC cell lines (AsPC-1, BxPC-3, Capan-1, and CFPAC-1). Nuclei stained with DAPI are blue. LAMP1 (green) was used as a lysosomal marker. Magnification, 60×. (**D**) Immunocytochemical detection of PEPT1 (green) in PEPT1-positive PDAC cell lines (AsPC-1, and SU.86.86). Nuclei stained with DAPI are blue. LAMP1 (red) was used as a lysosomal marker. Magnification, 60×. Data are given as mean ± SEM. * *P* < 0.05, ** *P* < 0.01, *** *P* < 0.001, **** *P* < 0.0001.

To test whether the expression of PEPT1 and PEPT2 correlated with transport function, we monitored the uptake of Gly-Sar, a hydrolysis-resistant dipeptide substrate for PEPTs, in these cell lines. Uptake buffers containing NMDG chloride in place of NaCl with two pH conditions (pH 5.5 and pH 7.5) were used to monitor H^+^-coupled Gly-Sar uptake. All PDAC cell lines, with the exception of BxPC-3, MIA PaCa-2, and PANC-1, demonstrated a significantly higher Gly-Sar uptake at pH 5.5 than at pH 7.5, providing evidence for H^+^-coupled dipeptide uptake (Figure 3A). As expected, normal cell lines did not exhibit H^+^-coupled Gly-Sar uptake. PEPT1 as well as PEPT2 are capable of H^+^-coupled dipeptide transport; therefore, the data in Figure 3A simply indicate the presence of PEPT transport function in PDAC cell lines but do not reveal whether the observed transport function is due to PEPT1 or PEPT2 or both. One of the revealing differentiating features of these two transporters is the difference in their substrate affinities. PEPT1 is a low-affinity transporter with almost a 10-fold less affinity for its substrates than PEPT2 for the same substrates. To check which PEPT transporter is functional in the PDAC cell lines, we performed Gly-Sar kinetics experiment in AsPC-1 cells to determine the kinetic parameters (Michaelis constant *K*_t_ and maximum velocity *V*_max_). If PEPT2 is functional in this cell line, then the *K*_t_ value should be in lower micromolar (0.005–0.5 µM) concentrations as opposed to PEPT1. Based on the kinetics experiment, the transport system was saturable and the uptake data fit well to a model describing a single saturable transport system. However, the values for *K*_t_ and *V*_max_ were 859.3 ± 22.46 µM and 8751 ± 22.46 pmol/mg protein/15 min, respectively, indicating PEPT1 and not PEPT2 to be functional in this cell line (Figure 3B). This was puzzling because PEPT2 protein is indeed expressed in these cells. To investigate as to why PEPT2 transport function was not detectable in the PDAC cell lines, we performed immunofluorescence studies to determine whether the PEPT2 protein localizes to the plasma membrane. For this, we used HPDE, AsPC-1, BxPC-3, Capan-1, and CFPAC-1 cells, which showed high level of PEPT2 protein expression. Surprisingly, the results showed that the majority of PEPT2 protein was localized in the nucleus with little evidence for its presence in the plasma membrane or lysosomes (Figure 3C). In contrast, immunofluorescence for PEPT1 in AsPC-1 and SU.86.86 showed majority of PEPT1 to be localized in the plasma membrane and lysosomes (Figure 3D). Since the technique of Gly-Sar uptake used in the present study monitors only the uptake across the plasma membrane, the immunofluorescence studies support the conclusion that PEPT1, not PEPT2, is solely responsible for the observed dipeptide uptake in the PDAC cell lines. While AsPC-1 had the highest PEPT1 protein expression, it did not have the highest PEPT functionality as shown by the Gly-Sar uptake. In fact, SU.86.86 maintained highest functionality. Next, Capan-1 despite having lower protein expression than AsPC-1 was functionally as good as AsPC-1 and in fact exhibited a very clean Gly-Sar uptake at pH 5.5 with almost a negligible uptake at pH 7.5. Therefore, SU.86.86 and Capan-1 were utilized for the majority of remaining experiments, including *in vivo* murine experiments. MIA PaCa-2 was used as a PEPT1-negative cell line.

### Glibenclamide inhibits PEPT1 function

**Figure 4.**
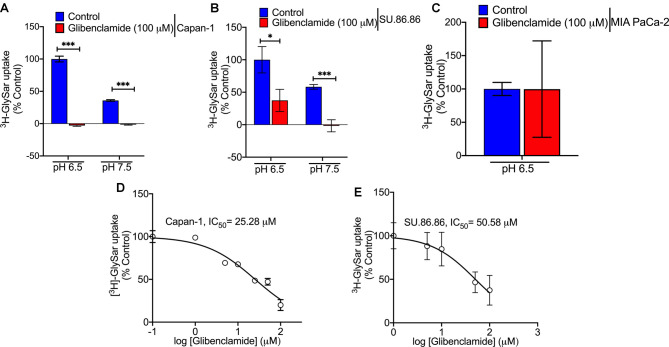
Glibenclamide inhibits PEPT1 function. (**A**–**C**) [^3^H]-Gly-Sar uptake in PEPT1-positive PDAC cell lines (Capan-1, SU.86.86) and PEPT1-negative PDAC cell line (MIA PaCa-2) in the presence or absence of glibenclamide at pH 6.5 and 7.5. (**D** and **E**) [^3^H]-Gly-Sar uptake showing dose-dependent inhibition by glibenclamide in PEPT1-positive Capan-1 and SU.86.86 PDAC cell lines. Data are given as mean ± SEM. * *P* < 0.05, *** *P* < 0.001.

Studies have already shown that glibenclamide, a sulfonylurea used for the treatment of non-insulin-dependent diabetes mellitus (type 2 diabetes), has the ability to inhibit PEPT1 and PEPT2-mediated Gly-Sar uptake with Ki values of 25 µM and 7.8 µM, respectively [27]. Therefore, to determine if glibenclamide could be used as a pharmacological agent to block the function of PEPT1 in PDAC cell lines, we monitored the effect of this compound on Gly-Sar uptake in Capan-1 and SU.86.86 (PEPT1-positive) and MIA PaCa-2 (PEPT1-negative) cells. [^3^H]-Gly-Sar uptake was determined in the presence of 100 µM glibenclamide at pH 6.5 and 7.5. Glibenclamide inhibited PEPT1-mediated Gly-Sar uptake completely in Capan-1 cells in both pH conditions (Figure 4A). The results were mostly comparable in SU.86.86 (Figure 4B). As PEPT1 is a H^+^-coupled transporter, the uptake function is detectable at pH 6.5 as well as at pH 7.5, and as expected, the uptake at pH 6.5 is greater than the uptake at pH 7.5. Glibenclamide had no effect on Gly-Sar uptake in MIA PaCa-2 cells (Figure 4C) because there is no PEPT1 in these cells and the uptake observed is minimal, mostly representing non-specific diffusional process. The dose-response study demonstrated that glibenclamide inhibited PEPT1-mediated [^3^H]-Gly-Sar uptake with an IC_50_ value of 25 µM and 50 µM in Capan-1 and SU.86.86, respectively. These IC_50_ values agree with the reported Ki values for PEPT1 in the literature [27].

### Glibenclamide inhibits the colony formation ability of PDAC cells

**Figure 5.**
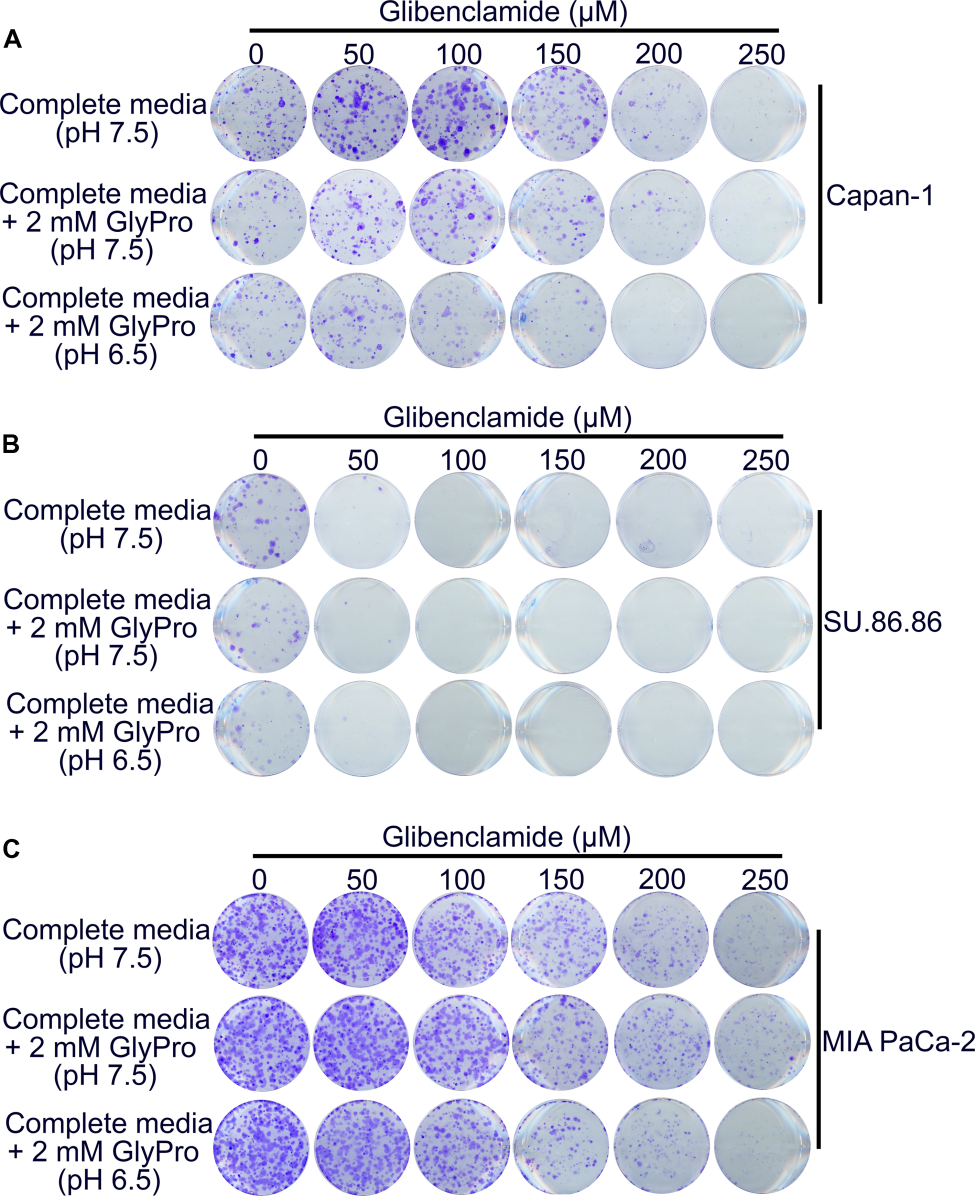
Glibenclamide inhibits colony formation ability of PDAC cells. (**A**–**C**) Colony formation assay in PEPT1-positive Capan-1 and SU.86.86 cells and PEPT1-negative MIA PaCa-2 cells. All three cell lines were cultured in the absence and presence of increasing concentrations of glibenclamide (0–250 µM) in three culture conditions i.e. complete medium at pH 7.5, complete medium at pH 7.5 plus 2 mM Gly-Pro, and complete medium at pH 6.5 plus 2 mM Gly-Pro.

Since glibenclamide inhibits PEPT1 function, we were curious to check whether glibenclamide has any impact on the cell biological features of PDAC cells. For this, PEPT1-positive (Capan-1 and SU.86.86) and PEPT1-negative (MIA PaCa-2) cells were cultured in the presence of increasing concentrations of Glibenclamide (0–250 µM) in different culture conditions: complete medium (pH 7.5), complete medium (pH 7.5) with 2 mM Gly-Pro, and complete medium (pH 6.5) with 2 mM Gly-Pro. The availability of substrate (Gly-Pro) and the H^+^-gradient (pH 6.5) is essential for PEPT1 to exert its effects on the cells. We hypothesized that if PEPT1 plays any role in the maintenance of amino acid nutrition in PDAC cells, glibenclamide should negatively impact cell proliferation and the effect would be maximal under the culture conditions where the substrate and the driving force are available for PEPT1. Our studies showed decreasing proliferation of Capan-1 cells with increasing concentrations of glibenclamide (Figure 5A); the effect was maximal in the presence of Gly-Pro and at pH 6.5. SU.86.86 cells showed a more robust sensitivity to glibenclamide than Capan-1 cells, with cell proliferation being significantly stunted in the presence of 50 µM glibenclamide (Figure 5B). MIA PaCa-2, though PEPT1-negative, also demonstrated a slight sensitivity to glibenclamide (Figure 5C); but the effects were comparable in all three culture conditions, indicating a PEPT1-independent mechanism for the observed effect. These data clearly suggested that glibenclamide by inhibiting PEPT1 had the ability to attenuate the growth of PDAC cells, thereby implicating that glibenclamide could be used as an anticancer agent for pancreatic cancer.

### Genetic knockdown of PEPT1 reduces the colony formation ability of PDAC cells

**Figure 6.**
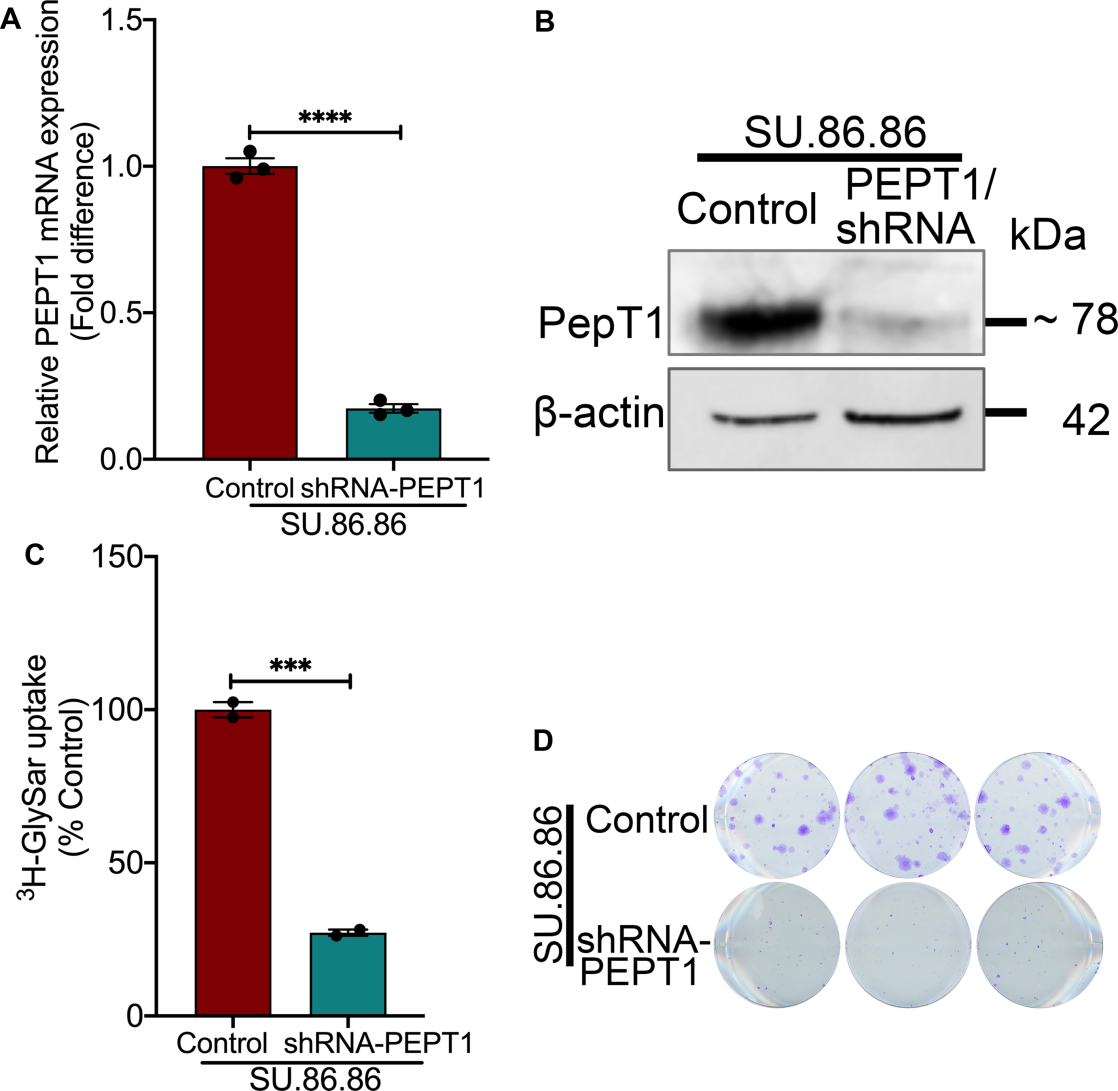
Genetic knockdown of PEPT1 reduces colony formation ability of PDAC cells. (**A**) Real-time PCR showing relative PEPT1 mRNA expression in control SU.86.86 cell line vs. the SU.86.86/shRNA-PEPT1 cell line. (**B**) Western blotting showing PEPT1 protein expression in control SU.86.86 cell line vs. the SU.86.86/shRNA-PEPT1 cell line. β-Actin was used as the endogenous control. (**C**) PEPT1-mediated [^3^H]-Gly-Sar uptake in control SU.86.86 cell line vs. the SU.86.86/shRNA-PEPT1 cell line. (**D**) Clonogenic assay in control SU.86.86 cell line vs. the SU.86.86/shRNA-PEPT1 cell line. Data are given as mean ± SEM. *** *P* < 0.001, **** *P* < 0.0001.

To test if genetic knockdown of PEPT1 would have similar effects as that of pharmacological inhibition, we used SU.86.86 cells as the model and silenced PEPT1 using PEPT1-specific shRNA. Among the five shRNAs tested, TRCN0000043299 (299, Millipore Sigma) demonstrated the most down-regulation of PEPT1 (∼83%), evident both at the transcriptional as well as translational levels (Figure 6A,B). We also found a corresponding decrease in Gly-Sar uptake (∼63%) in shRNA-cells (Figure 6C). We then examined whether the genetic deletion of PEPT1 would also affect its proliferation capacity. The colony-formation assay using SU.86.86/WT and SU.86.86/shRNA-PEPT1 clearly showed that PEPT1 loss does significantly impact the growth of SU.86.86 cells (Figure 6D).

### Pharmacological inhibition and genetic knockdown of PEPT1 decrease the growth of PDAC cells in mouse xenografts

**Figure 7.**
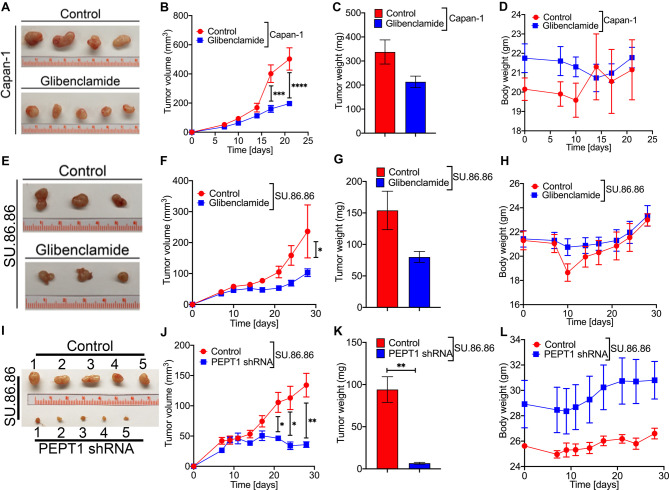
Pharmacological inhibition and genetic knockdown of PEPT1 decrease the growth of PDAC cells in mouse xenografts. Capan-1 and SU.86.86 cells were subcutaneously implanted in athymic nude mice. Representative photographs of harvested tumors from control and glibenclamide-treated Capan-1 cells (**A**) and SU.86.86 cells (**E**). Tumor growth curves of Capan-1 (**B**) and SU.86.86 (**F**) tumor-bearing nude mice treated with vehicle or glibenclamide. Tumor weights between control and glibenclamide-treated Capan-1 (**C**) and SU.86.86 cells (**G**). Evaluation of mouse body weights during the xenograft experiment in Capan-1 (**D**) and SU.86.86 (**H**). Subcutaneous xenograft of control SU.86.86 cells and SU.86.86/shRNA-PEPT1 in athymic nude mice showing representative images of harvested tumors (**I**), tumor growth curves (**J**), tumor weight (**K**), and mice body weight (**L**) between control and PEPT1-knockdown groups. Data are given as mean ± SEM. * *P* < 0.05, ** *P* < 0.01, *** *P* < 0.001, **** *P* < 0.0001.

To assess whether PEPT1 could be used as a drug target to treat pancreatic cancer *in vivo*, mouse xenograft studies were performed in athymic nude mice using PEPT1-positive Capan-1 and SU.86.86 as the model cell lines. A week after the subcutaneous implantation of the cancer cells, the mice were divided into control and treatment groups. The treatment group was treated with 166 mg/kg glibenclamide by oral gavage for 21 (Capan-1) and 28 (SU.86.86) days. Glibenclamide treatment led to a significant reduction in tumor growth with both Capan-1 and SU.86.86 cells, as evident from changes in tumor volume (Figure 7A,B,E,F) and the tumor weight (Figure 7C,G). The body weight of the mice did not change following glibenclamide treatment (Figure 7D,H), suggesting no obvious toxicity of glibenclamide. To investigate whether shRNA-mediated knockdown of PEPT1 would have similar effects, SU.86.86/WT and SU.86.86/shRNA-PEPT1 cells were injected subcutaneously in athymic nude mice and followed for 30 days. PEPT1 knockdown led to a robust reduction in tumor size (6.7 ± 0.1 mg) compared with untreated controls (94.1 ± 10.2 mg) (Figure 7I–K). Again, there was no significant change in body weight due to treatment (Figure 7L).

### Relevance of tumor-derived lactic acid in the TME to the transport function of PEPT1

**Figure 8.**
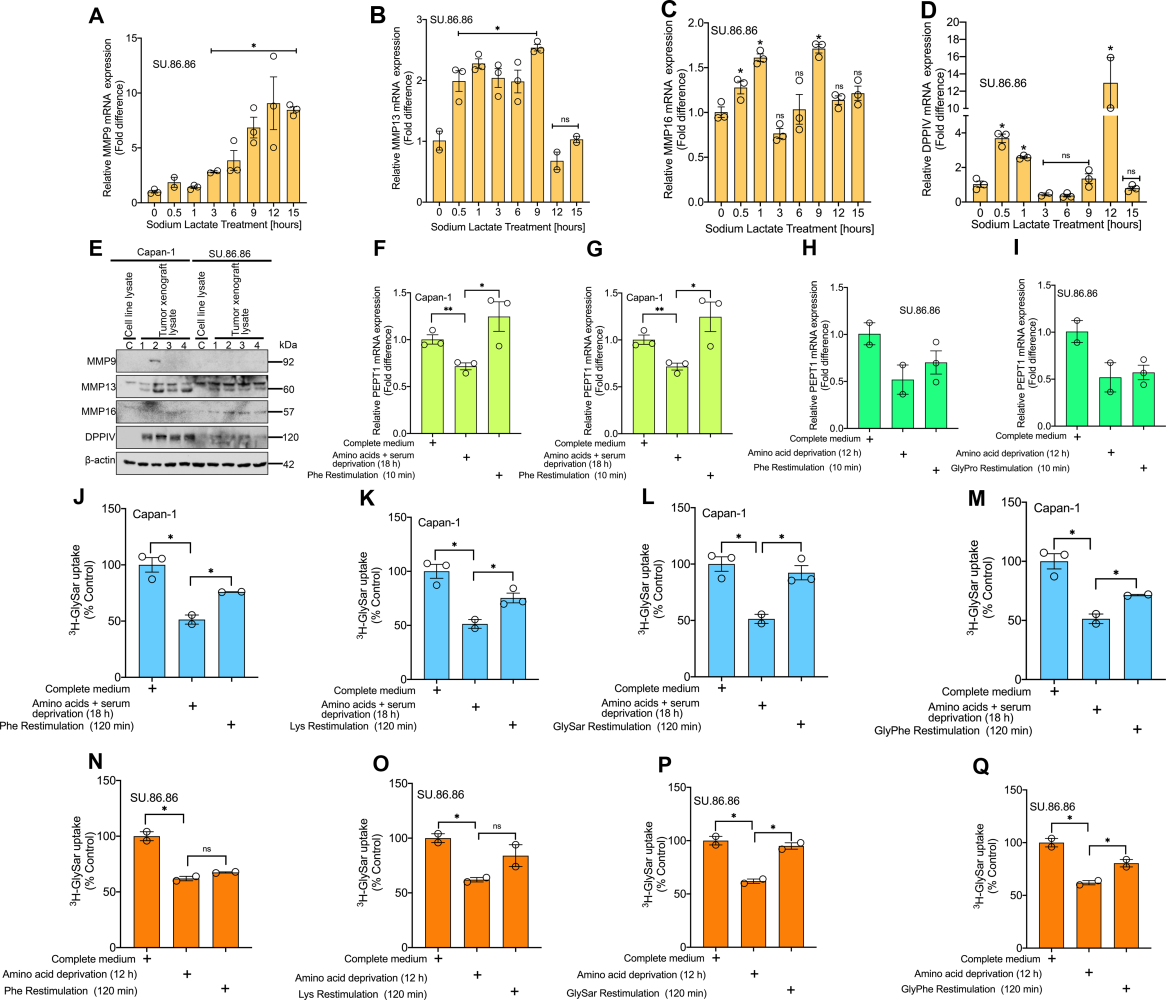
Lactate in the TME as a possible signaling molecule in support of PEPT1 function. Real-time PCR showing relative mRNA expression of MMP9 (**A**), MMP13 (**B**), MMP16 (**C**), and DPPIV (**D**) in control and lactate-treated SU.86.86 cells. Western blotting showing protein expression of MMP9, MMP13, MMP16, and DPPIV from total cell lysates of cultured Capan-1 and SU.86.86 vs. the tumor lysates from Capan-1 and SU.86.86 xenografted tumors. Beta-actin was used as a loading control (**E**). Real-time PCR showing relative PEPT1 mRNA expression in amino acid- and serum-starved Capan-1 and SU.86.86 cells restimulated with Phe (**F** and **G**) and Gly-Pro (**H** and **I**). PEPT1-mediated [^3^H]Gly-Sar uptake in amino acid- and serum-starved Capan-1 and SU.86.86 cells restimulated with Phe (**J** and **N**), Lys (**K** and **O**), Gly-Sar (**L** and **P**), and Gly-Phe (**M** and **Q**). Data are given as mean ± SEM. * *P* < 0.05, ** *P* < 0.01, *** *P* < 0.001, **** *P* < 0.0001.

The TME in PDAC is highly desmoplastic and consists of the immune cells, pancreatic stellate cell-derived myofibroblast-like cells, and ECM with collagen. Of relevance to PEPT1 is collagen, which upon its proteolytic breakdown could generate di- and tripeptides as substrates for PEPT1 *in vivo*. MMPs in the TME would hydrolyze collagen into large peptides, which can be further hydrolyzed into dipeptides by DPPIV. The unique amino acid composition of collagen [Gly/Pro/Ala-X)_n_] is the most appropriate for the action of DPPIV which prefers peptides containing the Gly-Pro/Ala sequence at the N-terminus to release Gly-Pro or Gly-Ala dipeptides. In addition, the TME is acidic, which will provide the driving force for PEPT1. Since tumor cells generate lactic acid and release into the TME, we asked whether lactate in the extracellular medium acts as a signaling molecule to influence the generation of peptide substrates for PEPT1. To address this question, we treated SU.86.86 cells with 10 mM lactate for various time points and examined the expression levels of MMPs and DPPIV. We found that lactate treatment led to a significant increase in the mRNA levels of MMP9, MMP13, and MMP16 (Figure 8A–C) and DPPIV (Figure 8D). Similar results were also seen in AsPC-1 cells (Supplementary Figure S1A–D). Our next question was then to check whether the tumors growing in the TME by virtue of its exposure to lactate in the extracellular medium would show an elevation in MMPs and DPPIV protein expression. To test that, Western blotting was performed in Capan-1 and SU.86.86 tumor xenograft samples. Total lysates prepared from cultured Capan-1 and SU-86.86 with no TME involvement was used as controls. Based on our Western data, cultured Capan-1 and SU.86.86 samples had none to negligible expression of the MMPs and the DPPIV, however, interestingly, the xenograft tumors from these cell lines clearly exhibited higher levels of MMP9, MMP13, MMP16, and DPPIV indicating a possible influence of the lactate in the extracellular medium acting as a signaling molecule in regulating the expression of these genes (Figure 8E). Next, since previous reports have shown the effect of peptides and amino acids in PEPT1 induction [28], we wanted to test whether a similar phenomenon operates in PDAC cells as well. To test this, we performed a deprivation and restimulation assay using Capan-1 and SU.86.86 cells as the model cell lines. Amino acid and serum deprivation for 18 h in Capan-1 significantly decreased PEPT1 mRNA expression. More interestingly, restimulation of the cells to the amino acid phenylalanine or the dipeptide Gly-Pro restored PEPT1 mRNA expression (Figure 8F,G). Similar results were also seen in AsPC-1 (Supplementary Figure S1E,F). That said, in SU.86.86 cells that underwent amino acid deprivation with 1% FBS medium (12 h) did show a down-regulation in PEPT1 mRNA expression, however, we did not see full restoration of PEPT1 expression following restimulation of the cells to the amino acid phenylalanine or the dipeptide Gly-Pro (Figure 8H–I). It is possible that for SU.86.86 a longer restimulation time is required as opposed to Capan-1. Likewise, we also performed [^3^H]Gly-Sar uptake in both the cell lines using similar deprivation conditions but a prolonged restimulation time of 2 h. It was interesting to see that the transport data corroborated with the Real-time PCR data for Capan-1 (Figure 8J–M). More interestingly, with 2 h of re-exposure time, SU.86.86 showed a better PEPT1 functional restoration (Figure 8N–Q). Even though lactate induced the expression of MMPs and DPPIV, it had no effect on PEPT1 expression.

## Discussion

Cancer cells reprogram their metabolism to support their rapid proliferation and growth. This metabolic change necessitates the need for increased supply of various nutrients to feed into these accelerated metabolic pathways. Therefore, starving the tumor cells of their essential nutrients seems a logical strategy to suppress their growth and hence a plausible therapeutic paradigm for the treatment of cancer. As most of these nutrients are water-soluble and cannot traverse the plasma membrane by simple diffusion, cancer cells up-regulate specific transporters to meet their increased demands for nutrients [28]. These transporters represent potential drug targets for cancer therapy. The recent emphasis on tumor-selective metabolic pathways such as glutaminolysis, reductive carboxylation, and one-carbon metabolism places particularly a special focus on amino acid transporters for development of a new class of anticancer therapeutics [7,8,29,30]. In addition to the amino acid transporters, peptide transporters also could make potential contributions to amino acid nutrition in cancer cells, but this aspect has received very little attention. This is surprising because the acidic pH in the TME is ideal to provide the necessary driving force for the peptide transporters due to their transport function coupled to a transmembrane H^+^ gradient [31–34]. One of the reasons for the relative lack of interest in the potential role of the peptide transporters in cancer is the fact that amino acids are present at several-fold higher concentrations than peptides in the circulation. This casts doubt as to the physiological relevance of the peptide transporters to amino acid nutrition in cancer cells. There have been some studies examining the therapeutic potential of peptide transporters, but the focus was on the exploitation of these transporters for the delivery of peptide drugs or non-peptide drugs in the form of peptide prodrugs [35,36]. Obviously, what has been overlooked is the possibility that the TME might contain small peptides at concentrations much higher than found in the circulation and that peptide transporters could actually play a significant role in the maintenance of amino acid nutrition in cancer cells *in vivo*. This provided the rationale for the present study to examine the role of peptide transporters in pancreatic cancer.

**Figure 9.**
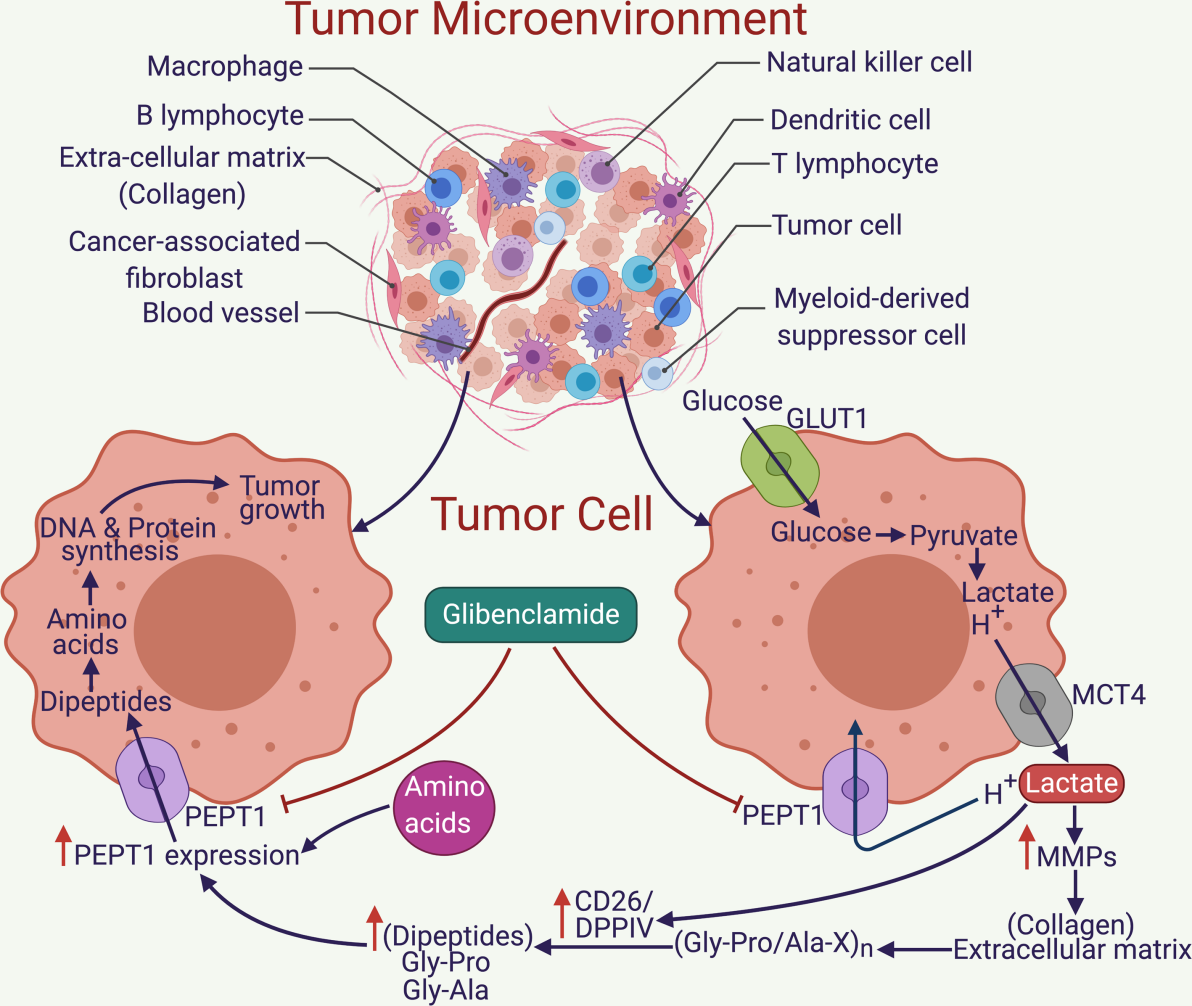
Graphical representation of how tumor-derived lactic acid can lead to the transcriptional activation of MMPs and DPPIV, which can degrade collagen within the extracellular matrix to di- and tripeptides; how these small peptides and the amino acids induce PEPT1 expression, which by the virtue of the acidic pH in the TME becomes functional and brings in dipeptide substrate inside the cancer cells, gets hydrolyzed into amino acids and helps in DNA and protein synthesis and ultimately tumor growth; and how pharmacological inhibition of PEPT1 with glibenclamide can starve the tumor cells of its substrates, eventually affecting its growth and proliferation.

The findings of the present study can be summarized as follows: (a) PEPT1 is markedly up-regulated in a majority of PDAC cell lines and PDXs; (b) PEPT1 is not just expressed at the mRNA and protein levels; it is actually functional and transport-competent in mediating the uptake of peptide substrates from the extracellular medium; (c) pharmacological inhibition and genetic knockdown with shRNA negatively affects the proliferation capacity of the cancer cells, both *in vitro* in cell culture as well as *in vivo* in mouse xenografts; (d) lactate in the TME functions as a signaling molecule to induce MMPs and DPPIV, which are likely to be involved in the proteolytic breakdown of extracellular collagen to generate di- and tripeptides as substrates for PEPT1; (e) amino acids and dipeptides in the extracellular medium induce the expression of PEPT1 in cancer cells. Furthermore, these findings provide a framework for the biological importance of the observed induction of PEPT1 in PDAC (Figure 9). These results demonstrate up-regulation of upstream markers after exposure to sodium lactate *in vitro* and tumor derived lactate *in vivo*, but no up-regulation in PEPT1 expression. While these results on their own do not seem to indicate PEPT1 regulation, previous research has demonstrated that PEPT1 has an amino acid response element (AARE) in the promoter region that causes transcriptional up-regulation of PEPT1 in response to available amino acids and dipeptides [28]. As current research understands various MMPs and DPPIV to cleave TME collagen into tripeptides, and further to dipeptides, this provides a stable framework through which PEPT1 up-regulation occurs in PDAC [37].

In a recent review, we speculated that the acidic pH in the TME might provide the driving force for a number of nutrient transporters whose function is coupled to a transmembrane H^+^ gradient [38]. These transporters include not only the peptide transporters but also the amino acid transporters in the SLC36 family, the folate transporter SLC46A1, and the iron transporter SLC11A2. The observations in the present study provide strong support for this notion with regard to the peptide transporter PEPT1.

The therapeutic potential of PEPT1 as a drug target in the treatment of PDAC has been validated in the present study using glibenclamide as a pharmacological inhibitor of the transporter. The efficacy of this drug as a potential anticancer agent is evident *in vitro* in cell culture and *in vivo* in xenografted tumor growth in mice. Multiple studies have already shown the anticancer property of glibenclamide *in vitro* in cell culture using cell lines representing cancer of the prostate [39], hepatocellular [40,41], breast [42], gastric [43], bladder [44], glioma [45], and colon [46]. But in these studies, it is either that the potential mechanisms underlying the anticancer efficacy of glibenclamide were not addressed or the focus was on the possible role of the voltage-gated K^+^ channel, the well-known molecular target for glibenclamide, in the observed anticancer effect. More importantly, none of these studies implicated PEPT1 in the pharmacological actions of glibenclamide. A recent study has shown that glibenclamide, by targeting the K^+^ channel, modulates the expression of p70S6K and KLF4 in non-small cell lung cancer, and exerts an anticancer effect in both *in vitro* as well as in *in vivo* models [47]. The present investigation confirms the anticancer efficacy of glibenclamide and also uncovers PEPT1 as a molecular target in this phenomenon. Obviously, inhibition of PEPT1 is not the sole mechanism for the ability of glibenclamide to inhibit tumor growth. The drug does decrease the growth *in vitro* of MIA PaCa-2 cells that are PEPT1-negative. Therefore, other molecular targets such as the K^+^ channel also play a role in this phenomenon.

Glibenclamide is used at a dose not more than 20 mg/day for the treatment of type 2 diabetes. In the present study, we found the drug to be effective in causing a significant reduction in tumor growth *in vivo* in mouse xenografts at a dose of 166 mg/kg/day. This translates into more than 1 g per day for humans with ∼70 kg body weight. However, even with this high dose, there was no evidence of toxicity seen in the mice used in the present study as evidenced by normal body weights compared with untreated control mice and the absence of drug-related morbidity. This is supported by a published report that glibenclamide did not lead to toxicity in mice even at a higher dose (200 mg/kg/day) [47]. It is not clear at present whether glibenclamide at this dose has any potential for cancer therapy in humans, but our studies at least serve as a proof-of-concept for development of more effective PEPT1 inhibitors for cancer treatment. While PEPT1 may be a viable target for pharmacological inhibition, its up-regulation in PDAC is similar to that of KRAS mutation percentage, with most, but not all, tumors expressing high levels of PEPT1. With this in mind, tumor composition and biopsies will still be extremely important in determining the correct course of treatment for patients with PDAC.

PEPT2 is also a H^+^-coupled transporter with higher affinity for peptide substrates than PEPT1. It is expressed in the plasma membrane in normal tissues such as the kidneys, lungs, and brain where it plays a role in the transport of peptides into cells [48,49]. In the present study we have shown that PEPT2 expression is markedly up-regulated in PDAC cell lines and PDXs. However, we were unable to find evidence for the expression of PEPT2 in the plasma membrane of the cancer cells; this is obvious from the lack of a high-affinity peptide transport activity in PDAC cell lines that are positive for PEPT2 mRNA and protein. The explanation for this conundrum comes from the findings that PEPT2 protein is localized in PDAC cells intracellularly. For the uptake of peptides from the extracellular medium into cells, the transporter must be present in the plasma membrane. This does not mean that the observed up-regulation of PEPT2 in pancreatic cancer is not relevant to tumor growth. The transporter could have some function in intracellular organelles that is still related to tumor biology. Many intracellular organelles have acidic pH in the lumen (e.g. lysosomes, Golgi) which provides the driving force for the peptide transporter to mediate the transfer of peptides from the lumen into the cytoplasm. Some of these organelles handle small peptides under physiological conditions. For example, lysosomes are involved in the breakdown of proteins. It is generally assumed that lysosomal proteolysis generates solely free amino acids, but there is no proof for this notion. With all the proteases in the intestinal lumen, digestion of dietary proteins produces predominantly small peptides and, only to a small extent, free amino acids [50,51]. There is no reason to believe that the lysosomal protein breakdown should be any different. Nuclear membrane also possesses a pH gradient with H^+^ concentration in the cytoplasm higher than in the nucleus, which might be of relevance to the findings in the present study that PEPT2 might be located in the nuclear membrane. The biological function of PEPT2 in this intracellular location and its potential connection to cancer cell biology remain to be determined.

In summary, our data suggests PEPT1 to have an essential role for pancreatic cancer cell growth and therefore could prove to be a viable drug target.

## Data Availability

All datasets generated and/or analyzed during the current study are included in this manuscript.

## Open Access

Open access for this article was enabled by the participation of Texas Tech University Health Sciences Center in an all-inclusive *Read & Publish* pilot with Portland Press and the Biochemical Society under a transformative agreement with EBSCO.

## Abbreviations

AARE: amino acid response element
CAC: colitis associated cancer
DPPIV: dipeptidyl peptidase IV, CD26
DSS: dextran sodium sulfate
ECM: extracellular matrix
FBS: fetal bovine serum
Gly-Phe: Glycyl-L-phenylalanine
Gly-Pro: Glycyl-L-Proline
Gly-Sar: Glycyl-Sarcosine
IBD: inflammatory bowel disease
MMP13: matrix metalloproteinase 13
MMP16: matrix metalloproteinase 16
MMP9: matrix metalloproteinase 9
NMDG: N-methyl-d-glucamine
PDAC: pancreatic ductal adenocarcinoma
PDX: patient-derived xenograft
PEPT1: peptide transporter 1, SLC15A1
PEPT2: peptide transporter 2, SLC15A2
PHT1: peptide/histidine transporter 1, SLC15A4
PHT2: peptide/histidine transporter 2, SLC15A3
PLP1: packaging lentiviral plasmid 1
PLP2: packaging lentiviral plasmid 2
TME: tumor microenvironment
VSVG: vesicular stomatitis virus G glycoprotein
