# Supplementary material for: PEPT1 is essential for the growth of pancreatic cancer cells: a viable drug target
Source: Biochem J. 2021 Oct 21;478(20):3757–74. doi: 10.1042/BCJ20210377 (PMC8589330; doi:10.1042/BCJ20210377)
Supplement: Supplementary Figure S1 [file BCJ-478-3757-s1.pdf]

Supplemental material

Figure legend

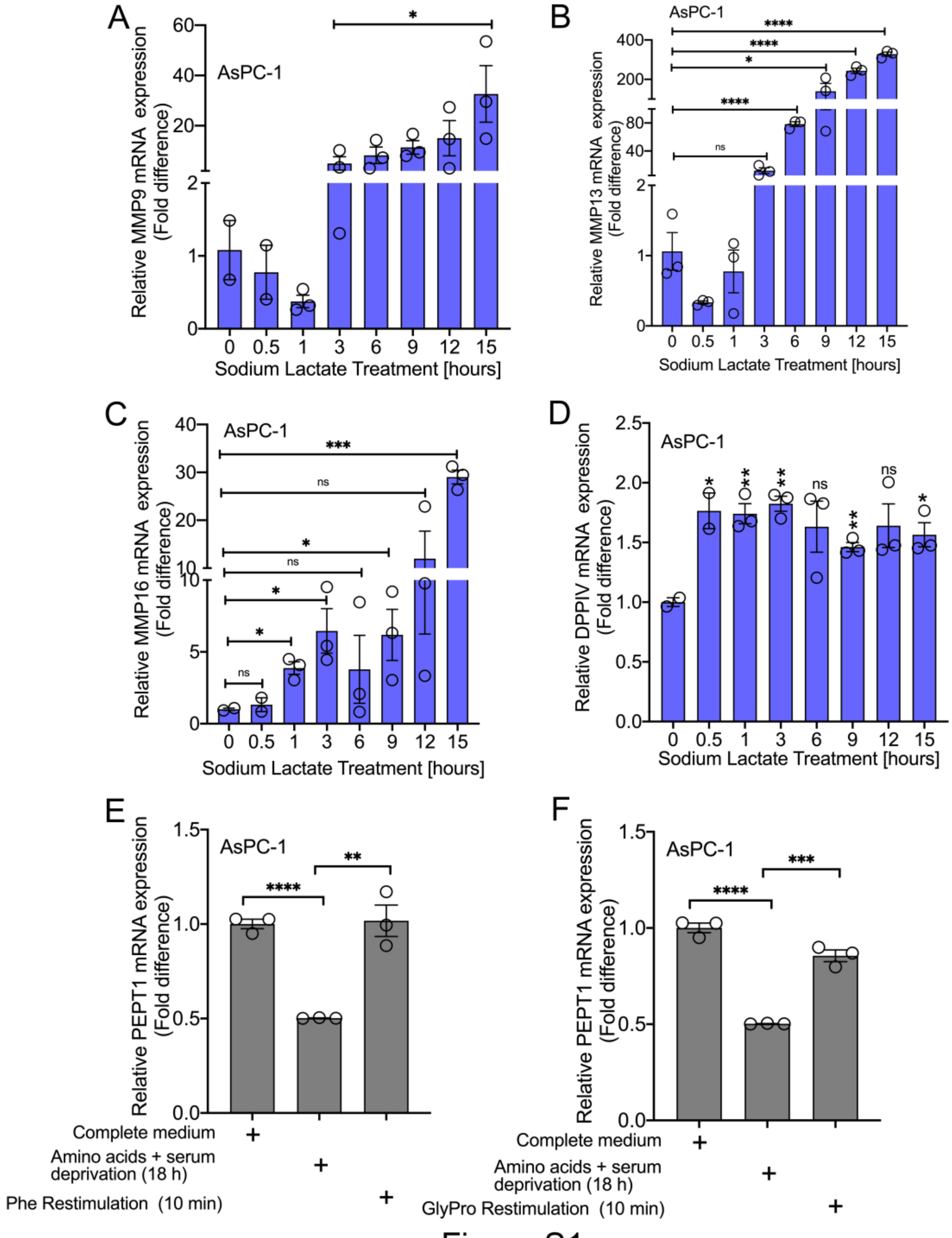

Figure S1

Figure S1. Lactate in the TME as a possible signaling molecule in support of PEPT1 function. Real-time PCR showing relative mRNA expression of MMP9 (**A**), MMP13 (**B**), MMP16 (**C**), and DPPIV (**D**) in control and lactate-treated AsPC-1 cells. Real-time PCR showing relative PEPT1 mRNA expression in amino acid- and serum-starved AsPC-1 cells re-stimulated with Phe (**E**) and Gly-Pro (**F**). Data are given as mean ± SEM. *$p<0.05$, **$p<0.01$, ***$p<0.001$, ****$p<0.0001$.
